# Public Perception of the Use of Digital Contact-Tracing Tools After the COVID-19 Lockdown: Sentiment Analysis and Opinion Mining

**DOI:** 10.2196/33314

**Published:** 2022-03-04

**Authors:** Zhilian Huang, Evonne Tay, Dillon Wee, Huiling Guo, Hannah Yee-Fen Lim, Angela Chow

**Affiliations:** 1 Department of Clinical Epidemiology Tan Tock Seng Hospital Singapore Singapore; 2 Nanyang Business School Nanyang Technological University Singapore Singapore; 3 Lee Kong Chian School of Medicine Nanyang Technological University Singapore Singapore

**Keywords:** infectious disease, sentiment analysis, opinion mining, COVID-19, contact tracing, public health, opinion, data mining, survey, cross-sectional

## Abstract

**Background:**

Singapore’s national digital contact-tracing (DCT) tool—TraceTogether—attained an above 70% uptake by December 2020 after a slew of measures. Sentiment analysis can help policymakers to assess public sentiments on the implementation of new policy measures in a short time, but there is a paucity of sentiment analysis studies on the usage of DCT tools.

**Objective:**

We sought to understand the public’s knowledge of, concerns with, and sentiments on the use of TraceTogether over time and their preferences for the type of TraceTogether tool.

**Methods:**

We conducted a cross-sectional survey at a large public hospital in Singapore after the COVID-19 lockdown, from July 2020 through February 2021. In total, 4097 respondents aged 21-80 years were sampled proportionately by sex and 4 age groups. The open-ended responses were processed and analyzed using natural language processing tools. We manually corrected the language and logic errors and replaced phrases with words available in the syuzhet sentiment library without altering the original meaning of the phrases. The sentiment scores were computed by summing the scores of all the tokens (phrases split into smaller units) in the phrase. Stopwords (prepositions and connectors) were removed, followed by implementing the bag-of-words model to calculate the bigram and trigram occurrence in the data set. Demographic and time filters were applied to segment the responses.

**Results:**

Respondents’ knowledge of and concerns with TraceTogether changed from a focus on contact tracing and Bluetooth activation in July-August 2020 to QR code scanning and location check-ins in January-February 2021. Younger males had the highest TraceTogether uptake (24/40, 60%), while older females had the lowest uptake (8/34, 24%) in the first half of July 2020. This trend was reversed in mid-October after the announcement on mandatory TraceTogether check-ins at public venues. Although their TraceTogether uptake increased over time, older females continued to have lower sentiment scores. The mean sentiment scores were the lowest in January 2021 when the media reported that data collected by TraceTogether were used for criminal investigations. Smartphone apps were initially preferred over tokens, but the preference for the type of TraceTogether tool equalized over time as tokens became accessible to the whole population. The sentiments on token-related comments became more positive as the preference for tokens increased.

**Conclusions:**

The public’s knowledge of and concerns with the use of a mandatory DCT tool varied with the national regulations and public communications over time with the evolution of the COVID-19 pandemic. Effective communications tailored to subpopulations and greater transparency in data handling will help allay public concerns with data misuse and improve trust in the authorities. Having alternative forms of the DCT tool can increase the uptake of and positive sentiments on DCT.

## Introduction

COVID-19, declared a pandemic by the World Health Organization in March 2020, is highly transmissible, with infections leading to deaths and severe illnesses [1]. Contact tracing has been a critical measure in curbing infectious disease transmissions [2]. However, conventional methods are time-consuming, labor-intensive, subject to recall biases, and unscalable during large-scale outbreaks, such as COVID-19 [3].

Digital contact-tracing (DCT) tools can potentially address the problem of scale during the COVID-19 pandemic by capturing device encounters via Bluetooth-enabled smartphone apps or wearable devices [4,5]. Studies have suggested that DCT tools can help to increase the detection of cases and reduce the time taken for contact tracing by 2.5 times [6-8]. However, a minimum population adoption rate of 60% is required for contact tracing to be effective with DCT tools [9]. At present, Singapore is the only country that has achieved a nationwide DCT adoption rate of more than 70% [10].

Singapore developed a national DCT tool—TraceTogether—in March 2020 and promoted its use after exiting a lockdown in June 2020. Since then, mandatory use of the TraceTogether smartphone app or wearable token for check-ins to enter public venues (such as shopping centers, grocery stores, restaurants, cinemas, schools, and hospitals) has been introduced to increase its adoption [11]. Although the adoption rate of TraceTogether increased from 40% in July to more than 70% in December 2020 [10], the adoption of TraceTogether may have been involuntary under mandatory conditions.

Privacy concerns, lack of trust in the government, and pessimistic views on the effectiveness of DCT tools were barriers against its adoption [12-14]. The plethora of reasons for the hesitancy to adopt DCT tools suggests complex sentiments among users, which may have been deep seated under mandatory conditions. Since the large-scale adoption of DCT tools is unprecedented, understanding the complex sentiments associated with its use would help policymakers to adjust implementation approaches. Traditional qualitative analyses can identify in-depth user perspectives on a topic but are usually confined to smaller samples of text due to their resource-intensive requirements. Natural language processing (NLP) tools are less sensitive in identifying nuances in text data but are able to process a large number of texts in a shorter time frame. Therefore, the use of NLP tools would be most suitable for the analysis of a large number of short texts without in-depth meanings [15].

Opinion mining and sentiment analysis have been performed widely to understand the public’s reaction and challenges faced during the COVID-19 pandemic [16,17]. These methods are useful for consolidating a large amount of information in a short period. For example, policymakers can study public sentiments on new events or COVID-19 measures and tailor public health communications to mitigate negative emotions arising from the event or measure [17]. An increasing number of studies have utilized short social media texts, such as Tweeter posts, to understand public sentiments on the COVID-19 pandemic [18,19]. Despite the benefits of opinion mining and sentiment analysis in rapidly garnering the public’s opinion, the data collected from online platforms, such as microblogs, social media, app store reviews, and online surveys, were biased toward the more privileged and technologically savvy individuals [20,21]. Overrelying on data collected from social media sites would omit the views of social groups that do not use these sites [22,23].

There is also a paucity of sentiment analysis studies on the usage of DCT tools. An Irish study analyzed the sentiments of DCT app reviews and found predominantly positive sentiments; however, the review focused on voluntary app users who may be more accepting of DCT tools [20]. Given the lack of studies on user perspectives on DCT tools and biases of the data sources used for opinion mining, we sought to understand the public’s knowledge of, concerns with, and sentiments on the use of DCT tools over time, as mandated by the authorities for pandemic control, across an extensive demographic and age distribution.

## Methods

### Study Design

We conducted a cross-sectional survey in the 2 busiest ambulatory clinics at the second-largest public hospital in Singapore, starting from 1 month after the nationwide COVID-19 lockdown in 2020. Data collection occurred over 8 months from July 2020 through February 2021 during patients’ or their caregivers’ visit to the clinic. Respondents from ages 21 to 80 years were sampled proportionately by sex and four 15-year age groups to cover the perspectives of digital natives and digital immigrants. We included only citizens and permanent residents of Singapore as this population would best fit the context of our study.

### Timeline of TraceTogether Events in Singapore

The use of TraceTogether was widely promoted after the COVID-19 lockdown in Singapore. The smartphone app was initially promoted to trace encounters with users in close proximity but was updated in June 2020 to collect personal identifiers for more effective contact tracing. The app is available in multiple languages, including Bengali, Burmese, Chinese, English, Hindi, Melayu, Tamil, and Thai. In July 2020, the token form of TraceTogether was made available to seniors who do not own smartphones. Each token weighs 15 g, with dimensions that are 62 mm long, 15 mm thick, and 45mm wide. From September to November 2020, the government made a series of announcements to promote the uptake of TraceTogether. All Singapore citizens were eligible to collect a free TraceTogether token to facilitate mandatory safe entry check-ins at all public venues [11], and social restrictions would be eased further if at least 70% of the population adopted TraceTogether [24]. In early January 2021, the Singapore police force used the TraceTogether data for criminal investigations under the Criminal Procedure Code (CPC) [25]. Clarifications were made when the act evoked a public outcry on personal data protection.

### Survey Instrument

We designed a 14-item survey questionnaire based on literature review and included questions on the status of digital device usage and willingness to use TraceTogether (pre- and postsharing on DCT tools). Three open-ended questions were used to determine respondents’ knowledge of TraceTogether, top three concerns with any DCT technology, and the reasons for their preference for the form factor of TraceTogether (refer to Multimedia Appendix 1 for the questionnaire).

### Data Collection

We trained all data collectors to ensure that the questionnaire was appropriately administered by the interviewer. Information on respondents’ perceptions of a DCT tool was collected using TraceTogether as an example. We then provided a 2-minute explanation on the purpose of DCT tools at the end of the survey and asked respondents again whether they would be willing to use a DCT tool and, if so, whether they preferred an app or a token and the reason for their choice. Demographic information was collected to perform segmented analyses.

This study focused on the open-ended responses from respondents, which included asking the respondents their thoughts on the purpose, data security, and usage of TraceTogether and their concerns with TraceTogether. The reasons for the choice of a smartphone app or token were also analyzed.

### Descriptive Statistics

We classified respondents into 4 age and sex categories (younger females, older females, younger males, and older males) and classified those who were above the age of 50 years as older adults and those 50 or under 50 years as younger adults. Mean and SDs were computed for age, while proportions were computed for other categorical variables, such as demographics, smartphone ownership, awareness of TraceTogether, willingness to use TraceTogether, and preference for its form factor. We also presented the bimonthly uptake rate of TraceTogether by age and sex to show the impact of the policy measures to boost uptake rates.

### Data Processing

The open-ended responses were manually processed to correct language and spelling errors. Abbreviations were written in full, and the informal and colloquial form of the English language was rephrased to the formal form. For example, “don’t like” was rephrased as “dislike” as “don’t” would likely be removed as a stop word, while “like” would be detected as a positive sentiment, although the phrase implies a negative sentiment. Important phrases on “knowledge of TraceTogether” were standardized 3-word phrases and analyzed as trigrams (refer to Table S1 in Multimedia Appendix 1), while other sections were analyzed as bigrams. The processed trigrams were subsequently replaced with a phrase that was closer to their original meaning before it was presented graphically (eg, the pre-processed phrase “Location unknown uncollected” was replaced with “Location data NOT collected” when presented graphically).

**Figure 1:**
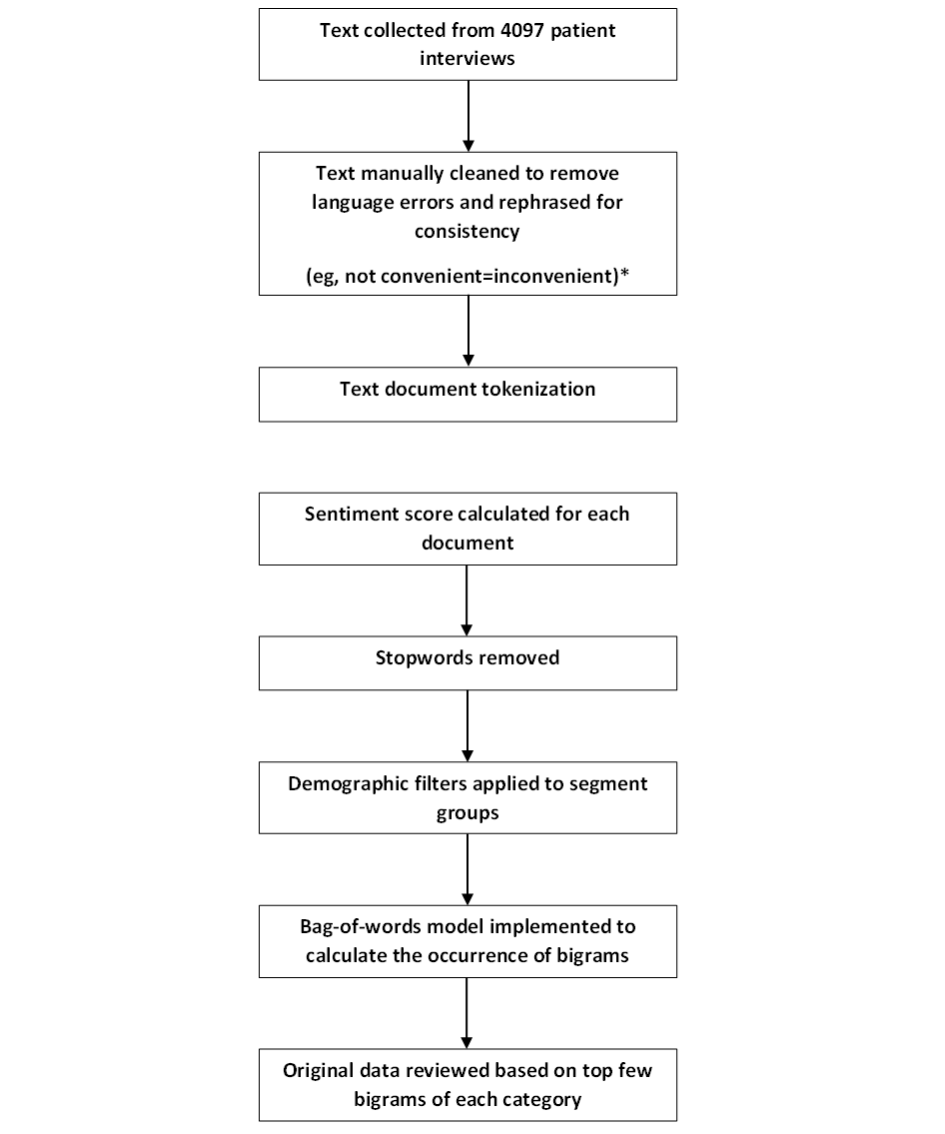
Process of data processing and analysis. *Refer to Table S1 and Figure S1 in Multimedia Appendix 1.

The preprocessed responses were then processed with NLP tools. All phrases were tokenized (split into smaller units), and the sentiment score of each phrase was computed by summing the sentiment scores of all the tokens in the phrase. Stopwords, such as prepositions and connectors, were removed according to the *stopwords* package in R, followed by implementation of the bag-of-words model (simplifying the representation of words) to calculate the occurrence of bigrams and trigrams in the data set. Demographic and time filters were applied to segment the responses, as required (Figure 1).

### Sentiment Analysis

We used the *syuzhet* package for sentiment analysis as it incorporates 3 other lexicons developed by other groups [26]. The *bing* lexicon contains a list of words classified into positive and negative sentiments, while the *nrc* lexicon classifies words into 8 other emotions on top of the positive and negative sentiments. The *afinn* and *syuzhet* lexicons contain a database of words with a sentiment score. We compared the sentiment scores derived from the *syuzhet* and *afinn* lexicon and did not find substantial differences in sentiment patterns [26,27]. Hence, we used the *syuzhet* lexicon for our analyses as the database contains a wider range of vocabulary compared to the *afinn* database. Each word in the *syuzhet* library has a value of between –1 and 1. Words with positive connotations are scored positively, while those with negative connotations are scored negatively. The sentiment score of a response statement was computed by summing the values of all the words in the statement that could be found in the *syuzhet* library. All analyses were performed using RStudio version 1.2.5033.

Some of the respondents would comment on the disadvantages of the TraceTogether token when asked why they preferred the smartphone app or vice versa. Hence, the reasons for respondents’ preference for either the TraceTogether tool (smartphone app or portable token) were split into token- or smartphone-related comments before sentiment analysis.

### Ethical Considerations

This study was approved by the National Healthcare Group (NHG) Domain Specific Review Board (DSRB) in Singapore (NHG DSRB ref. 2020/00775). A waiver of written informed consent was granted, and implied consent was assumed if the individual agreed to respond to the survey.

## Results

### Recruitment Rate and Demographics of Respondents

We approached 6260 potential respondents and excluded 744 (11.88%) who did not meet the inclusion criteria. Of 5229 eligible participants, we interviewed 4097 (78.35%) respondents in total. Approximately a quarter of the respondents who were interviewed declined to respond to the open-ended questions.

Table 1 shows the demographics of respondents. Age and sex were proportionately sampled during data collection. Hence, respondents were divided into 4 age and sex categories. The Chinese race was slightly oversampled in older adults as the Chinese race constitutes about 76% of the Singapore population. A higher proportion of younger adults were tertiary educated compared with older adults, but the overall education level of respondents was representative of the general population. A smaller proportion of older adults were employed, as 796 (38.81%) of 2051 older adult respondents were retirees.

**Table 1:** Baseline characteristics of respondents (N=4097).

Characteristics			Total respondents		Younger males		Younger females		Older males		Older females
**Age (years), mean (SD)**			50.2 (16.8)		35.4 (8.7)		35.7 (8.5)		64.8 (7.9)		64.7 (7.9)
**Ethnicity, n/N (%)**											
	Chinese	3330/4097 (81.27)		802/1024 (78.32)		756/1022 (73.97)		867/1027 (84.42)		905/1024 (88.38)	
	Malay	315/4097 (7.69)		107/1024 (10.45)		148/1022 (14.48)		49/1027 (4.77)		50/1024 (4.88)	
	Indian	354/4097 (8.64)		84/1024 (8.20)		84/1022 (8.22)		91/1027 (8.86)		56/1024 (5.47)	
	Others	98/4097 (2.39)		31/1024 (3.03)		34/1022 (3.33)		20/1027 (1.95)		13/1024 (1.27)	
**Education level, n/N (%)**											
	Tertiary	1296/4097 (31.63)		488/1024 (47.66)		489/1022 (47.85)		214/1027 (20.84)		105/1024 (10.25)	
**Employment status^a^, n/N (%)**											
	Employed	2728/4097 (66.59)		870/1024 (84.96)		872/1022 (85.32)		548/1027 (53.36)		438/1024 (42.77)	
**Digital device usage^b^, n/N (%)**											
	Owns a smartphone	3712/4097 (90.60)		1022/1024 (99.80)		1019/1022 (99.71)		871/1027 (84.81)		800/1024 (78.13)	
	Heard of TraceTogether	3818/4097 (93.19)		987/1024 (96.39)		972/1022 (95.11)		934/1027 (90.94)		925/1024 (90.33)	
	Willing to use TraceTogether (presharing)	3143/4097 (76.71)		739/1024 (72.17)		767/1022 (75.05)		806/1027 (78.48)		831/1024 (81.15)	
	Willing to use TraceTogether (postsharing; 36 [0.9%] missing)	3674/4061 (90.47)		908/1021 (88.93)		924/1018 (90.77)		903/1010 (89.41)		939/1012 (92.79)	
**Form factor preference (willing to use TraceTogether postsharing; 127 [3.5%] missing), n/N (%)**											
	Smartphone app	1900/3547 (53.56)		604/872 (69.27)		602/887 (67.87)		399/872 (45.76)		295/916 (32.21)	
	Token	1647/3547 (46.43)		268/872 (30.73)		285/887 (32.13)		473/872 (54.24)		621/916 (67.79)	

^a^Full-time, part-time, self-, or temporary employment.

^b^Uptake of TraceTogether was not presented in this table, as uptake rates changed over time with the policy measures. Refer to Figure 2 for bimonthly uptake rates.

**Figure 2:**
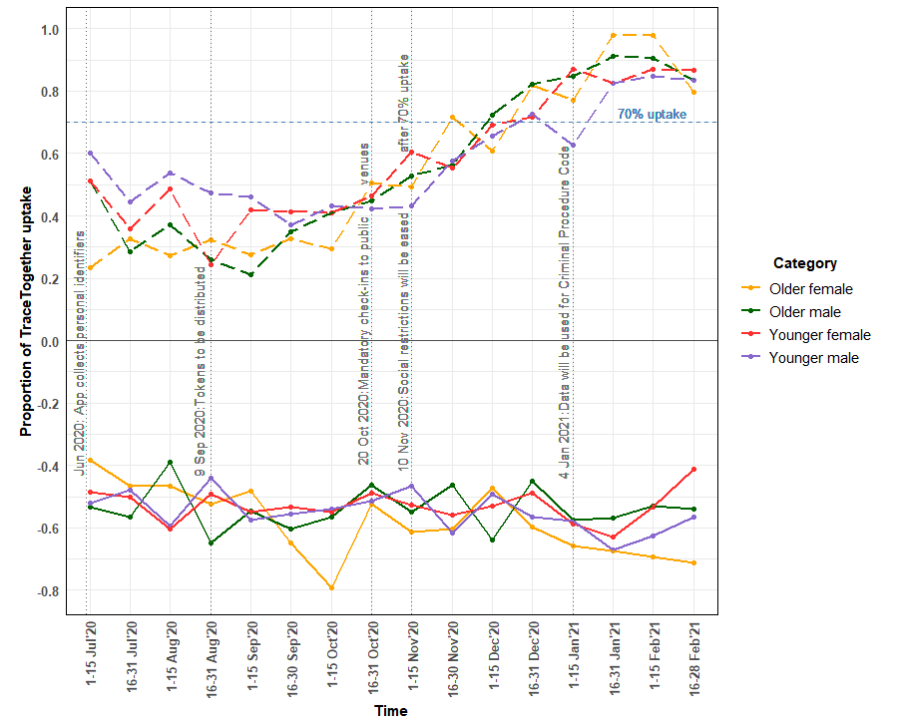
The proportion of TraceTogether uptake and mean (syuzhet) sentiment score of concerns with TraceTogether over time.

The overall smartphone ownership was 3712 (90.60%) of 4097 people. However, the proportion of older adults who owned a smartphone was lower than that of younger adults. Older females had the lowest smartphone ownership compared with other groups. The majority of respondents (3818/4097, 93.19%) had heard of TraceTogether at the time of the survey. The willingness to use TraceTogether increased across all age and sex categories after the study team explained the rationale and benefits of TraceTogether to the respondent.

### Knowledge of TraceTogether

**Figure 3:**
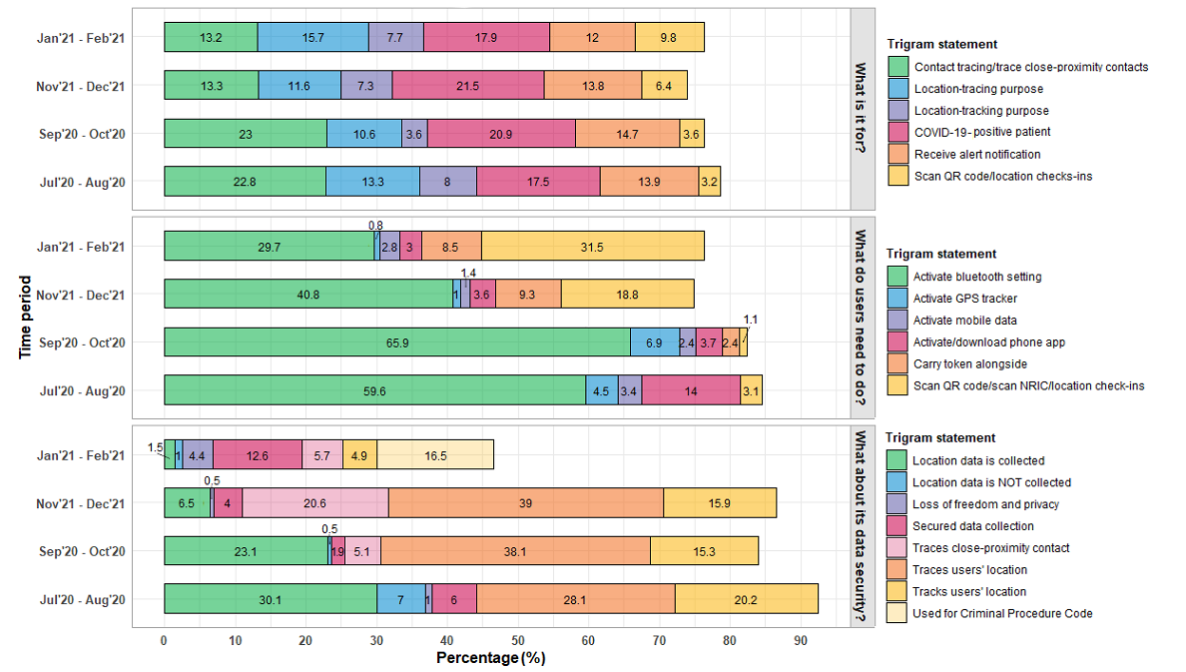
Participants’ responses on their knowledge of TraceTogether changed over time. The occurrence of trigrams and their proportions relative to all trigrams in a time category were computed. GPS: Global Positioning System; NRIC: National Registration Identity Card. *Trigrams were rephrased for clarity.

We counted the occurrence of unique trigrams and further collapsed trigrams with similar meanings to reduce the number of statements. Figure 3 shows the proportion of top trigrams classified into 2-month periods. All but 1 period had trigrams covering at least 70% of responses. Overall, the proportion of trigrams, representing respondents’ knowledge and perceptions of TraceTogether, changed over time.

The top 6 trigrams from respondents’ opinion on the purpose of TraceTogether (“What do you think TraceTogether is for?”) were “Contact-tracing purpose/trace close-proximity contacts,” “Location-tracing purpose,” “Location-tracking purpose,” “COVID-19-positive patient,” “Receive alert notification,” and “Scan QR code/location check-ins.” The proportion of mentions on contact tracing decreased (from 414/1806 [22.92%] to 365/2755 [13.25%]), while mentions on QR code scanning and location check-ins increased (from 19/588 [3.23%] to 133/1362 [9.77%]) over time.

The top 6 trigrams from respondents’ opinion on the usage of TraceTogether (“What do you think users need to do?”) were “Activate Bluetooth setting,” “Activate GPS tracker,” “Activate mobile data,” “Activate/download phone app,” “Carry token alongside,” and “Scan QR code/scan NRIC/location check-ins” (where NRIC stands for National Registration Identity Card). Initially, respondents thought they had to download the app (41/292, 14.04%) and activate Bluetooth on their smartphones (174/292, 59.59%) to use TraceTogether. Over time, the proportion of mentions on Bluetooth activation decreased from 174 (59.59%) of 292 to 284 (29.71%) of 956, while the proportion of mentions on scanning QR codes/location check-ins increased from 9 (3.08%) of 292 to 301 (31.49%) of 956. The proportion of mentions on the need to carry the TraceTogether token also increased from 0 in August 2020 to 81 (8.47%) of 956 in December 2020.

When respondents were asked their opinion on the data security of TraceTogether, three-quarters of the responses from July to October 2020 were mentions of “location data collected” or “users’ location traced/tracked.” By December 2020, the proportion of mentions related to location tracking/tracing/data collection decreased to 263 (61%) of 428 and subsequently to 25 (6.44%) of 388 by February 2021. The proportion of mentions on “secured data collection” doubled from 18 (5.96%) of 302 in July-August 2020 to 49 (12.63%) of 388 in November-December 2020. However, 64 (16.49%) of the 388 trigrams in January-February 2021 were mentions of the use of data collected by TraceTogether for the “Criminal Procedure Code.”

### Concerns With the Use of TraceTogether

We present the proportion of TraceTogether uptake (question item on “Are you currently using the TraceTogether app or Token?”) and the mean *syuzhet* sentiment score of concerns with TraceTogether (open-ended question on “Please list your top three [3] main concerns with any DCT technology [not limited to TraceTogether]”) at 2-week intervals in Figure 2. The plots were segmented into 4 categories: older males, older females, younger males, and younger females. Older adults were aged between 51 years and 80 years, while younger adults were between 21 years and 50 years of age. Uptake rates increased rapidly after the announcement of mandatory TraceTogether check-ins at public venues in mid-October 2020 and reached 70% by December 2020. The magnitude of the sentiment scores was used to compare sentiment changes over time, and the concerns with TraceTogether were negative overall.

Younger males had the highest TraceTogether uptake (24/40, 60%), while older females had the lowest uptake (8/34, 24%) in the first half of July 2020. This trend was reversed in mid-October after the announcement on mandatory TraceTogether check-ins at public venues. In mid-February 2021, the TraceTogether uptake of older adults surpassed 90% (93/99), while that of younger adults was 80%-90% (100/116).

The mean sentiment scores were the lowest in January 2021 when the media reported that the data collected by TraceTogether were used for criminal investigations. Older females also had decreased sentiment scores as their TraceTogether uptake increased over time. This group of respondents were mainly concerned about data breaches, privacy violation, and the pressure to adopt a new technology that they were unfamiliar with. Four respondents had misconceptions about the Bluetooth technology and cited health concerns about possible radiation emitted by TraceTogether. Younger adults had similar concerns with privacy violation but were also concerned about Bluetooth battery consumption on their smartphones.

We present the occurrence of bigrams on respondents’ concerns with the usage of TraceTogether in Table 2. The bigrams were classified into 5 categories. Each bigram was presented with a corresponding example of a response statement and the sentiment score of the statement.

**Table 2:** Top bigrams (N=3995) of respondents’ concerns with TraceTogether.

Bigram		Occurrence, n (%)	Example of response statement	Sentiment score of example statement
**TraceTogether app** **inconvenience**				
	Battery drainage	489 (12.24)	*Bluetooth needs to be activated, which causes battery drainage and slows down the app.* [Male, 57 years, September 2020]	–0.25
	Technical glitch	83 (2.08)	*Technical glitch. It malfunctions on my phone all the time and does not seem to capture any interactions that I made with the people around me. That is why I stopped utilizing it.* [Female, 41 years, August 2020]	–1.5
	Phone battery	77 (1.93)	*Inconvenient. If [my] phone battery [goes] flat, it [TraceTogether] would not work anymore.* [Female, 57 years, November 2020]	–0.25
	User unfriendly	22 (0.55)	*App is user unfriendly.* [Female, 49 years, October 2020]	–0.5
	Bluetooth battery	22 (0.55)	*Bluetooth battery drainage.* [Respondents aged 21-71 years, both sexes, all months]	–0.25
	Memory space	10 (0.25)	*Lack of phone memory storage space.* [Female, 27 years, July 2020]	–0.75
	Phone memory	9 (0.23)	*Lack of phone memory storage space.* [Female, 27 years, July 2020]	–0.75
	Language barrier	5 (0.13)	*Language barrier. [The] Interface is in English. The first page should especially be in mandarin as 70% of the population is Chinese and some may not understand [English].* [Male, 68 years, July 2020]	–0.5
**TraceTogether app and token data security**				
	Privacy violation	386 (9.66)	*Data privacy violation. Even though they [the authorities] say they collect minimal data, you would not know if they changed it down the road since nobody reads the terms and conditions.* [Male, 46 years, November 2020]	–0.5
	Data privacy	238 (5.96)	*Location data privacy violation. You do not want others to know some places you go to.* [Female, 68 years, January 2021]	–0.5
	Dislike location	139 (3.48)	*Linked with location tracking and location tracing*	—^a^
	Location tracking	96 (2.40)	*Dislike location tracking. The app will track my location like an ankle collar on criminals.* [Male, 54 years, December 2020]	–1.5
	Location tracing	53 (1.33)	*Dislike location tracing. Mistrust the government on data safety.* [Female, 57 years, November 2020]	–1.2
	Data insecurity	81 (2.03)	*Lack of transparency on data usage, concerned about data insecurity.* [Male, 29 years, July 2020]	–2.1
	Data leak	66 (1.65)	*Afraid of [data] being hacked and having data leak.* [Male, 27 years, September 2020]	–2.1
	Data unprotected	9 (0.23)	*Data [are] unprotected. Dislike having to input IC [identification] number. Phone number should suffice.* [Male, 68 years, September 2020]	–1.8
**TraceTogether app and token data misuse**				
	PDPA^b^ violation	152 (3.80)	*PDPA violation. Dislike that personal details have to be entered before you can utilize it.* [Female, 42 years, December 2020]	–1.5
	Data breach	148 (3.70)	*Who is responsible if there is a data breach and how is the breach being handled?* [Female, 50 years, July 2020]	–0.5
	Privacy invasion	41 (1.03)	*Privacy invasion. A sense that you are being stalked.* [Male, 67 years, July 2020]	–1.4
	Jeopardize bank details	17 (0.43)	*Jeopardize bank and credit card details.* [Respondents aged 26-67 years, both sexes, September 2020-February 2021]	–0.5
	Credit card details	16 (0.40)	*Jeopardize bank and credit card details.* [Respondents aged 26-67 years, both sexes, September 2020-February 2021]	–0.5
	Personal information	18 (0.45)	*Privacy violation as there may be a personal information leak.* [Female, 47 years, January 2021]	–0.85
	Information leak	12 (0.30)	*Privacy violation as there may be a personal information leak.* [Female, 47 years, January 2021]	–0.85


**TraceTogether app and token efficiency and efficacy of contact tracing**				
	Contact tracing	58 (1.45)	*Even with contact tracing we are unable to immediately know who is infected or if I met them, so it is inefficient.* [Female, 40 years, February 2021]	–1.35
	TraceTogether inaccurate	6 (0.15)	*TraceTogether [is] inaccurate. It is pointless if I utilize and others do not.* [Male, 50 years, October 2020]	–1.5
	Delayed notification	6 (0.15)	*The app notified me of possible exposure on the 14th, but I received delayed notification only on the 30th.* [Male, 30 years, December 2020]	–0.15
**TraceTogether token design**				
	Size cumbersome	35 (0.88)	*The token is badly designed, has a limited lifespan and its large size [is] cumbersome.* [Male, 74 years, December 2020]	–2
	Token size	12 (0.30)	*Cumbersome token size and it is unaccepted at some stores.* [Female, 51 years, December 2020]	–0.5

^a^Not applicable.

^b^PDPA: Personal Data Protection Act.

#### Inconvenience Created by the TraceTogether App

In this study, 717 (17.95%) of 3995 bigrams were concerns with the inconvenience created by the TraceTogether app. The concerns included smartphone battery drainage due to Bluetooth activation, frustration with technical glitches, and the app taking up phone memory space. Older individuals may have language barriers with using the app, and individuals with dependents may find the process of checking into locations troublesome.

Bluetooth needs to be activated, which causes battery drainage and slows down the app.

Inconvenient. If [my] phone battery [goes] flat, it [TraceTogether] would not work anymore.

User unfriendly. Will appreciate [it] if you can include your child in the parents’ app as well so can save time on scanning as the child's details are inside the parents' app.

Language barrier. [The] interface is in English. The first page should especially be in [M]andarin as 70% of the population is Chinese and some may not understand [English].

#### Concerns With Data Security in the TraceTogether App and Token

In this study, 1068 (26.73%) of 3995 bigrams were concerns with the data security of TraceTogether. Respondents disliked location tracking or tracing and felt that their (data) privacy was/will be violated with the use of TraceTogether. Respondents also felt that data transparency was insufficient, and they were insecure about data leaks should they lose their token.

Data privacy violation. Even though they [the authorities] say they collect minimal data, you would not know if they changed it down the road since nobody reads the terms and conditions.

Location data privacy violation. You do not want others to know some places you go to.

Dislike location tracking. The app will track my location like an ankle collar on criminals.

Dislike location tracing. Mistrust the government on data safety.

Lack of transparency on data usage, concerned about data insecurity.

#### Concerns With Data Misuse by the TraceTogether App and Token

In this study, 404 (10.11%) of 3995 bigrams were concerns with data misuse, such as violation of the Personal Data Protection Act (PDPA), data breaches, and leakage of personal information and credit card details. Respondents also felt that tagging the TraceTogether device provided a sense of privacy invasion and insecurity about possible data breaches.

PDPA violation. Dislike that personal details have to be entered before you can utilize it.

Who is responsible if there is a data breach and how is the breach being handled.

Privacy invasion. A sense that you are being stalked.

#### Concerns With the Efficiency and Efficacy of Contact Tracing

In this study, 70 (1.75%) of 3995 bigrams were mentions of the accuracy of TraceTogether and delayed notifications. Respondents were concerned that TraceTogether would be inaccurate if most of the population does not utilize TraceTogether appropriately. Some respondents mentioned that they did not receive timely app notifications of possible exposures.

Inefficiency of the tool. The success of contact tracing depends on the cooperation of citizens.

The app notified me of possible exposure on the 14th (of the month), but I received delayed notification only on the 30th (of the month).

#### Dissatisfaction With the TraceTogether Token Design

In this study, 47 (1.18%) of 3995 bigrams were mentions of dissatisfactions with the TraceTogether token form factor. Respondents felt that the large token size makes it cumbersome to carry around. Other issues related to the token include its limited battery span, unsightly aesthetics, and inability to check in at smaller stores without token scanners.

The token is badly designed, has a limited lifespan, and its large size [is] cumbersome.

Cumbersome token size and it is unaccepted at some stores.

### Preference for the TraceTogether Tool

**Figure 4:**
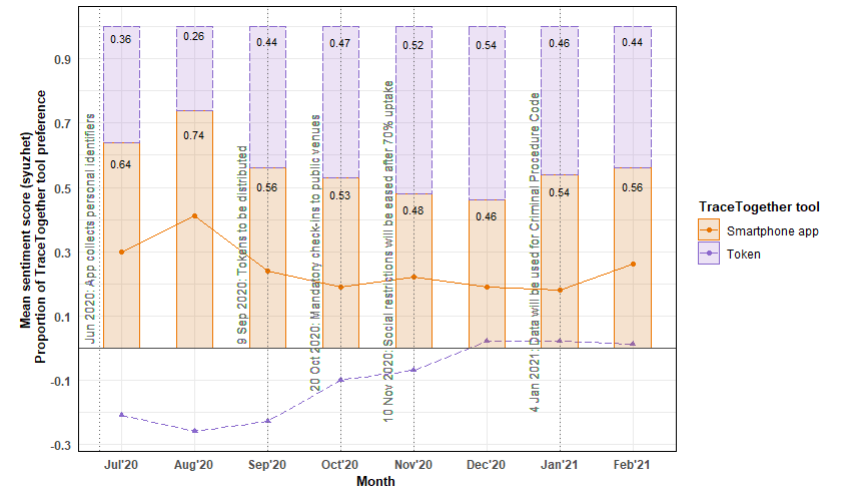
The proportion of respondents’ preference for the TraceTogether tool (smartphone app or token) and the sentiment scores of the reason for their choice over time. Note: The proportion of TraceTogether preferences are based on cross-sectional time series data.

Respondents’ preference for the type of the TraceTogether tool (smartphone app or portable token) and sentiment scores of the reason for their preferred type over time are shown in Figure 4. In the first 2 months of data collection, in July and August 2020, two-thirds (96/150, 64%) and three-quarters (161/217, 74.2%) of respondents preferred the smartphone app over the token. Over time, the preference for the type of TraceTogether tool equalized among respondents as tokens became accessible to the whole population.

The sentiment scores of the reason for the preferred type of TraceTogether tool moved in tandem with the proportion of respondents’ indicating preference for that particular type. The sentiments on token-related comments became more positive as the preference for tokens increased. Similarly, the sentiments on smartphone app–related comments became less positive as the preference for the smartphone app decreased. Overall, respondents had more positive sentiments on the use of the TraceTogether app compared with the token.

Respondents preferring the smartphone app felt that the app was a convenient option since they would always have their phones with them. Respondents preferring the app also felt burdensome to have to remember to bring the token when going out and were worried that the token, if misplaced, would be misused by the finder. In addition, some respondents commented that the plastic used to manufacture the tokens was environmentally unfriendly and that the tokens were unsightly and bulky to carry. There were also smaller shops that did not allow the checking-in of tokens.

Respondents preferring the token felt that the tokens were suitable for the elderly with difficulty using smartphone apps. They liked that the token does not consume the smartphone’s battery and mobile data and that there is no need to charge the token or worry about their smartphone battery going flat.

## Discussion

### Principal Findings

We found differences in the sentiments of respondents across age, sex, and time under voluntary and mandatory use of TraceTogether. The application of NLP techniques on unstructured free-text responses to open-ended questions in an interviewer-administered questionnaire implemented consistently over 8 months enabled us to quantify and examine the changes in public sentiments as the COVID-19 pandemic evolved. Such analyses will provide useful and timely feedback to policymakers on the impact of public health policies and measures imposed on the population and enable them to fine-tune them for greater public acceptance and compliance.

The knowledge of TraceTogether did not differ across age groups. Respondents’ knowledge of the purpose of TraceTogether did not change substantially over time, except for a slight shift from its use for contact tracing to scanning of QR codes for location check-ins. A small proportion of respondents (450/5206, 8.64%) had the misconception that TraceTogether tracks their location. Polls have shown that 1 reason for the hesitancy in TraceTogether uptake is the misconception that TraceTogether tracks the user’s location [28]. More could be done by the authorities to address such misconceptions in public education.

Respondents’ perception of the security of the data collected by TraceTogether shifted from being location focused from July to December 2020 to a focus on the Criminal Procedure Code and loss of freedom and privacy in January-February 2021. The overall sentiments on the concerns with TraceTogether were most negative in January 2021. Despite the negative sentiments on the usage of TraceTogether data for the CPC, there was also a higher proportion of mentions of secured data collection in January-February 2021, implying improved knowledge of and confidence in TraceTogether’s data handling among some respondents. The media is powerful in eliciting knee-jerk reactions among the public. Although such reactions may be short lived, it is imperative to promptly address any public concern to prevent long-term repercussions [29].

The sentiments on concerns with TraceTogether became more negative after the government’s announcement on the mandatory use of TraceTogether for check-ins to enter public venues, although the reported uptake rate of TraceTogether increased. This observation suggests involuntary uptake of TraceTogether required for social interactions to resume. In another study, we assessed the trade-offs of social interactions and incentives on the use of DCT tools and found that most people would prefer to use a DCT tool in exchange for more social interactions under conditions of social restrictions during the COVID-19 pandemic.

The involuntary uptake of TraceTogether may lead to negative sentiments due to a lack of understanding of the data transparency and the need for users to tag their identifiers with the tool. Although the negative sentiments could be transient and existent only during the COVID-19 pandemic, prolonged negative population sentiments may lead to future political repercussions if the benefits of the mandatory measures are unappreciated [30].

The sentiments and preference for TraceTogether tokens improved after mass token distributions to the public. Although smartphone apps were preferred, having an alternative type of TraceTogether tool could have improved the overall uptake of TraceTogether. Reducing barriers to accessibility may have helped to increase the TraceTogether uptake rate as no other country has nationally distributed alternative forms of the DCT tool or achieved more than 70% uptake [31].

### Limitations

There are various limitations to this study. First, the observations were cross-sectional, as we did not assess opinion changes of the same respondents over time. Nonetheless, the serial cross-sectional surveys on proportionately sampled respondents with a good representation of age and sex at every period provided invaluable insights into the changes in the population’s opinions over time. Second, the *stopwords* package removed too many words, while the existing sentiment libraries did not have sentiment scorings for colloquial phrases such as “don’t like,” “don’t want,” and “not friendly.” We had to manually replace these words with words found in the sentiment library to apply a sentiment score to the phrase. Regardless, the meaning of the words was retained. Third, manual data cleaning is time-consuming and may not be feasible for analyzing a large data set in a short amount of time. Lastly, respondents were patients and visitors of a public hospital and may not be representative of the Singapore population. However, a good representation of sex and age groups were sampled.

Future studies could explore crowdsourcing to develop a sentiment library that better suits the local context to reduce the time spent on data preprocessing. A stop word library that excludes (does not remove) words used in colloquial phrases will also reduce efforts on data preprocessing. Timely awareness of the public’s sentiments on a new policy will allow policymakers to adjust their approach to public communication [32]. The insights gained from subpopulations, such as the elderly and adults who are not technologically astute, provide opportunities to tailor interventions that can help them to better adapt to the new technology.

### Conclusion

In conclusion, the public’s knowledge of and concerns with using a mandatory DCT tool varied with the national regulations and public communications over time with the evolution of the COVID-19 pandemic. Effective communications tailored to subpopulations and greater transparency in data handling will help allay public concerns with data misuse and improve trust in the authorities. Having alternative forms of the DCT tool can increase the uptake of and positive sentiments on DCT tools.

## Appendix

Multimedia Appendix 1 Additional information on data processing and questionnaire.

## Abbreviations

CPC: Criminal Procedure Code
DCT: digital contact tracing
NRIC: National Registration Identity Card
PDPA: Personal Data Protection Act
