# Supplementary material for: Public Perception of the Use of Digital Contact-Tracing Tools After the COVID-19 Lockdown: Sentiment Analysis and Opinion Mining
Source: JMIR Form Res. 2022 Mar 4;6(3):e33314. doi: 10.2196/33314 (PMC8900919; doi:10.2196/33314)
Supplement: Multimedia Appendix 1 [file formative_v6i3e33314_app1.docx]

**Supplementary files**

**Questionnaire used for the study**

| **STATUS OF DIGITAL DEVICE USAGE** | | |
| --- | --- | --- |
| **Q** | **Question** | **Options** |
| 1 | Do you own a smartphone? | Yes/No |
| 2 | Have you heard of the TraceTogether app or Token? |  |
| 3 | Are you currently using the TraceTogether app or Token? | No/ Yes – app only/ Yes – token only/ Yes – both app and token |
| 4 | Can you describe to me what you know about the TraceTogether app or Token? (Probe for its functionality, data privacy, and what they think users need to do) | Open ended |
| **WILLINGNESS TO USE TRACETOGETHER** | | |
| **Q** | **Question** | **Options** |
| 5a | I am willing to use the TraceTogether app | Agree/Not agree |
| 5b | I am willing to use the TraceTogether token |  |
| 5c | I am willing to use both the TraceTogether app and token |  |
| 6 | Please list your top three (3) main concerns with any digital contact tracing technology (not limited to TraceTogether) | Open ended |
| **DEMOGRAPHICS** | | |
| **Q** | **Question** | **Options** |
| 7 | What is your age (in years) according to your last birthday? | Integer only |
| 8 | What is your gender? | Male/Female |
| 9 | What is your ethnicity? | Chinese/ Malay/ Indian/ Eurasian/ Others |
| 10 | What is the highest level of education you have attained? | No formal education / Primary education/ Secondary education/ Post-Secondary education (or vocation)/ Diploma/ Degree/ Postgraduate Qualification (Masters or PhD) |
| 11 | What is your employment status? | Full-time permanent/ Part-time permanent/ Temporarily employed  Self-employed/ Unemployed/ Retired |
| **POST-SHARING ON DIGITAL CONTACT TRACING TOOLS** | | |
| **Q** | **Question** | **Options** |
| 12 | Are you willing to use a digital contact tracing tool similar to TraceTogether app or Token? | Yes/No |
| 13 | If Q13 “Yes”, which form of a digital contact tracing tool would you prefer? | Smartphone app/ Token |
| 14 | What is/are the reason(s) for your choice in Q14 | Open ended |

**Table S1: List of standardized trigrams from the knowledge of TraceTogether section**

| **Standardized words** | **Phrase in text** | **Interpretation** |
| --- | --- | --- |
| **Activate Bluetooth setting** | Bluetooth exchange; Bluetooth needed; Bluetooth signal; Bluetooth technology; Enable Bluetooth; On Bluetooth; Switch on Bluetooth; Turn on Bluetooth; Use Bluetooth; Uses Bluetooth |  |
| **Activate GPS tracker** | GPS; Location services |  |
| **Activate mobile data** | Data; Internet; Mobile network |  |
| **Activate phone APP** | Activate APP |  |
| **Activate Wi-Fi internet** | Wi-Fi |  |
| **Carry token alongside** | Bring (Take) token out; Leave it in bag; Put token in the bag when going out; Leave token in bag |  |
| **Check-in check-out location** | Check into; Entry; Exit; For entry; In; Out; To go in and out of one place |  |
| **Close proximity contact** | Around; Beside; Close by contact; Close contact; Detect nearby contact  Near; Near where you are; Nearby people; People in proximity; Surrounding; Trace surrounding; people; Vicinity; Who you have been with | Refers to close proximity contact with TraceTogether users in the context. Replaced with “Traces close-proximity contact” in the final output. |
| **Contact tracing purpose** | Contact trace; Contact tracing; Trace contact; Tracing contact |  |
| **Criminal Procedure Code** | CPC; Police use data for investigation | Replaced with “Used for Criminal Procedure Code” in the final output |
| **Detect collect location** | Know your location; Location detected; Location known | Replaced with “Location data is collected” in the final output |
| **Download phone app** | Download application; Install |  |
| **Exchange phone signal; Exchange Bluetooth signal** | Detect nearby phone; Exchange Bluetooth; Exchange interactions; Exchange phone signal; Exchange TraceTogether |  |
| **Location tracing purpose** | Logs place you have been to; Trace location; Trace where you go |  |
| **Location tracking purpose** | Monitor where you go; Track your movements; Track location; Where I am, time and place |  |
| **Location unknown uncollected** | No location; Not detected; Not tracked | Replaced with “Location data NOT collected” in the final output |
| **Loss (of) freedom (and) privacy** | Govt track us; Loss of freedom; Loss of privacy; Sense of privacy and freedom taken |  |
| **Personal information collected** | Phone number recorded; NRIC |  |
| **Positive COVID-19 patient** | Coronavirus; Contagious; COVID; COVID/COVID-19 patient(s); COVID-19 case; Someone got COVID-19; Positive case; Confirmed case; Infected; Infectious; With virus |  |
| **Receive alert notification** | Alert(ed); Call up; Contacted; Informed; Notification; Notified; Prompt; Update | Interpreted in the context of the question and responses |
| **Register activate Singpass** | Singpass | Singpass is a digital identity for Singapore citizens and permanent residents to perform transactions with government agencies online. |
| **SafeEntry token or APP** | Scan entry |  |
| **Scan NRIC barcode** | IC; Identity card; NRIC; Scan NRIC |  |
| **Scan QR code** | QR code; QR scanning; Scan;  Scan code; Scan QR; Scan with it |  |
| **Secured data collection** | Confidential; Data secured; Need to upload data to government when contacted and passcode was given; Not intruded; only collected from positive patient; Only government knows the data; Only MOH will use data if I upload; Only when I approve; Password;  Private; Safe; Trust in no misuse |  |
| **Suspected COVID-19 patient** | Close contact infected; People may be infected |  |
| **Tap (the) device (against the) machine** | Just tap; Tap against machine; Tap on the machine | Refers to TraceTogether entries to public malls |
| **Timed data removal** | Remove data after 14 days; Data will be removed automatically |  |
| **Trace COVID-19 patient** | Trace positive patients only |  |
| **Unsecured data collection** | Data collected not secure; Data will leak; Not safe; Privacy compromised |  |
| **Unsure** | Don't know; No idea; Not sure; Unclear |  |

Rephrase query and emphasise on the uncertainty

(*Refer to Annex A*)

Elaborate on the response and standardise words

(*Refer to Annex B*)

Amend & ensure to maintain the original meaning and sentiments

No

Match the word to a word from the Syuzhet sentiment library

(*Refer to Annexes C & D*)

Replace Stop words with synonym as far as possible

(*Refer to Annex E*)

No

No

No

**Free text response**

**Is the response comprehensible?**

**Is the response phrased as an answer?**

**Is the response written in a complete sentence?**

**Is the response properly expressed in the English language?**

Yes

**Is the word available in the Syuzhet sentiment library?**

Yes

**Is the response free of Stop words?**

**Convert to standardised phrases for analysis**

(*Refer to Annex F*)

**Response is ready for analysis**

Yes

Yes

Yes

Yes

No

**Figure S1**: Flow chart of data processing the free text responses of “concerns with TraceTogether”

**Annex A: Selected rephrased responses that were answered as a question**

| **Phrases extracted from responses** | **Processed response phrases** |
| --- | --- |
| Accuracy of the TraceTogether app? | Uncertain if TraceTogether app is inaccurate |
| Data security is uncertain | Uncertain if personal data is unsecured |
| How accurate are the data collected | Uncertain if data collected is inaccurate |
| How convenient | Uncertain if it is inconvenient to utilize |
| How far has privacy protection laws been developed | Uncertain if privacy protection laws are undeveloped |
| How long will it be used for, even after the pandemic.? | Uncertain how long it will be utilized for and will it be utilized even after the pandemic |
| How safe is my personal data | Uncertain if personal data is unsafe |
| How safe it is (in term of privacy) | Uncertain if it is unsafe when it comes to privacy |
| How secure is the data collected | Uncertain if data collected is unsecured |
| How the data are being used? | Uncertain on how data is being utilized |
| If the app has access to other content in the phone | Uncertain if the app can access another phone content |
| In case of a network issue, how will they continue to collect data | Uncertain if data collection will be interrupted by a network issue |
| Law protection is to what extent if the data breached | Uncertain if the law will leave individuals unprotected during a breach of personal data |
| Not so sure how effective it is | Uncertain if it is ineffective |
| Not sure how data is traced, collected and who has access to it | Uncertain how data is traced, collected and who has access to it |
| The laws that protect us as individuals when using this type of app | Uncertain if laws leave us unprotected when using this type of app |
| What are they using the data for? | Uncertain of what the data is being utilized for |
| What data is being collected (what identifiable information) | Uncertain what identifiable information is being collected |
| What data is used for | Uncertain what data is utilized for |
| Whether it is accurate in tracking | Uncertain if TraceTogether is inaccurate in tracking |
| Whether my personal information will be breached | Uncertain if there will be a breach of personal information |
| Whether or not it is safe and if people can steal your data | Uncertain if it is unsafe and if data can be stolen |
| Whether or not there is secrecy of information. Government needs to tell us more details. | Uncertain if information is kept secret. Government needs to provide more information |
| Whether they know our exact location | Uncertain if they know our exact location |
| Will it leak my personal data | Uncertain if it will cause a personal data leak |
| Will location or data being collected be leaked | Uncertain if location or data collected will be leaked |

**Annex B: Selected one-word responses pre-processed to a phrase**

| **Original one-word responses** | **Pre-processed phrase** |
| --- | --- |
| Accuracy | TraceTogether inaccurate |
| Bank information | Breach of banking information |
| Battery | Battery drainage |
| Bothersome | Bothersome to utilize |
| Complicated | Complicated to utilize |
| Confidentiality | Lack of confidentiality |
| Inaccurate | TraceTogether inaccurate |
| Inconvenient | Inconvenient to utilize |
| Ineffective | TraceTogether ineffective |
| Location | Location data privacy violation |
| Loss of PDPA/PDPA | PDPA violation |
| Personal data concerns | Data breach |
| Pointless | Pointless to utilize |
| Privacy | Privacy violation |
| Security | Data unsecured |
| Time consuming | time consuming to utilize |
| Troublesome | Troublesome to utilize |

**Annex C: Selected stop word matches to words in the Syuzhet library**

| **Negative phrases with stop words** | **Word available in the Syuzhet library** |
| --- | --- |
| Doesn't work | Malfunction |
| Don't feel/see/find the need/No need | Unnecessary |
| Don't know | Unaware |
| Don't understand | Misunderstand |
| Don't/Not remember | Forget |
| Lack of compatibility | Incompatible/incompatibility |
| Lacks efficiency/ Not very efficient | Inefficient |
| No discipline | Undisciplined |
| No freedom | Restricted |
| No idea | Clueless |
| No point | Pointless |
| Not (be) secured/ Not enough security | Unsecured |
| Not (so) effective | Ineffective |
| Not (so) reliable | Unreliable |
| Not (so) useful | Useless |
| Not a big fan/ Don't like | Dislike |
| Not accepted | Unaccepted |
| Not accurate | Inaccurate |
| Not aware | Unaware |
| Not convenient | Inconvenient |
| Not durable | Fragile |
| Not educated | Uneducated |
| Not enough | Insufficient |
| Not familiar | Unfamiliar |
| Not practical | Impractical |
| Not sure | Unsure |
| Not used to | Unaccustomed |

**Annex D: Selected flipped phrases to indicate a concern on TraceTogether**

| **Phrase to be flipped** | **Flipped word/phrase** |
| --- | --- |
| Accurate/accuracy | Inaccurate |
| Compatibility/compatible | Incompatible |
| Convenience/convenient | Inconvenient |
| Data protection | Data unprotected |
| Data Security/ Security of (personal) data or information | Personal data/information is unsecured |
| Effectiveness | Ineffective |
| Efficient/efficiency | Inefficient |
| Have to be easy to use/ Easy to use | Difficult to utilize |
| I worry about how safe the data is | I worry that the data is unsafe |
| It must be accurate and everyone must use if not it defeats the purpose | It must not be inaccurate…(continue) |
| Just make sure it is not difficult to utilize | It must not be difficult to utilize |
| Must be user friendly | Must not be user unfriendly |
| Not many are tech savvy | Many are unfamiliar with technology |
| Should be more user friendly | Is user unfriendly |
| Token should be more user friendly | Token should be less user unfriendly |
| User friendly especially for elderly. | Must not be user unfriendly for elderly |

**Annex E: Selected flipped phrases to indicate a concern on TraceTogether**

| **SMART Word (Stop word)** | **Replaced word** |
| --- | --- |
| Issue | Problem |
| Issues | Problems |
| Mishandling | Mishandle |
| On (when used to refer to something being activated) Switch on (when used to refer to something being activated) | Activated |
| Use (when used to refer to the function or goal of an entity) | Purpose |
| Use/used | Utilize |

**Annex F: Selected standardized phrases of concerns with TraceTogether**

| **Original Phrase** | **Pre-processed phrase** |
| --- | --- |
| No concern | No concerns |
| Effectiveness (not everyone is using it); Effectiveness as it relies on other people using it; Effectiveness of app depends on number of ppl using it; Effectiveness of contact tracing technology unless everyone uses it; Effectiveness of it (only a certain percentage of people are using it); Effectiveness of TT depends on the number of ppl using it | (tool) may be/is ineffective as/unless/if (reason) |
| Bank and credit card details compromised | Jeopardize bank and creditcard details |
| Affect the battery life span; Affects battery life; Battery consumption/consuming; Battery draining; Battery dying; Battery life(span); Battery usage; Depletes the battery; Depleting phone battery; Drain(s) (the) battery (life); Draining of battery; Draining power; Eats up battery life; Runs out my battery; Take up extra battery life; Uses a lot of battery power; Waste battery | Battery drainage* (does not apply when the token is explicitly mentioned) |
| Breach of info; Data breach; Personal data breach | Data breach |
| Data/info (being) leaked/leakage; Leakage of data | Data leak |
| (Personal) Data privacy | Data privacy violation |
| I am concerned about how safe my data is | Data unsafe |
| Hard to understand | Difficult to understand |
| Ease of operation/ use | Difficult to utilize |
| Bulky; Can be less big; Size is big; Too big in size; Very big | Large size cumbersome |
| Location privacy | Location data privacy violation |
| Being traced by location; Trace location; Trace my location | Location traced |
| Location tracking/tracked; Track my location; Tracking of location; Tracking our location; Tracks location | Location tracked |
| App space; App takes up a lot of storage space; Consume memory space; Memory space on phone; Storage space/usage; Takes up storage and capacity on phone | Memory space drainage |
| Data (internet) usage; Data consumption/usage; Eat(s) data; Usage of data plan; Uses a lot of internet data; Uses mobile data | Mobile data drainage |
| Privacy (being) invaded | Privacy invasion |
| No privacy; Personal privacy; Privacy issues/ concerns/ problem; Privacy not there | Privacy violation |
| Foreign to technology; Not tech savvy | Unfamiliar with (the) technology |
